# Vertical Aerosol Structure Matters: Improving the AOD–PM_2.5_ Link for Air Quality and Exposure

**DOI:** 10.1021/acs.est.6c00095

**Published:** 2026-05-08

**Authors:** Irina Rogozovsky, Albert Ansmann, Alexandra Chudnovsky

**Affiliations:** † Department of Geophysics, Raymond and Beverly Sackler Faculty of Exact Sciences, Air-O lab, 26745 Tel Aviv University, Tel Aviv 6997801, Israel; ‡ 28397 Leibniz Institute for Tropospheric Research, Leipzig 04318, Germany

**Keywords:** pollyXT-lidar, AOD, aerosol layering, machine learning, particulate matter

## Abstract

Relating satellite-derived aerosol optical depth (AOD) to surface PM_2.5_ is a primary challenge in global air quality assessment, hindered by variable vertical conditions and aerosol physicochemical properties. By conditioning five years of observational data on 10 distinct layering types, we demonstrate that the AOD-PM_2.5_ relationship is strongly regime-dependent. We identified specific conditions, such as deep dust with overlying anthropogenic layers, where column-to-surface coupling is robust (up to *R* ^2^ ≈ 0.7 under optimal configurations). Conversely, mixed-layer regimes exhibit physical decoupling, rendering the satellite signal nonrepresentative (*R* ^2^ ≈ 0). For elevated transport layers, the satellite signal often acts as a temporal precursor; “previous-day” lag variance outperforms concurrent data due to downward dust mixing. Lidar-derived relative humidity profiles further differentiate the boundary layer from the free troposphere, highlighting the effect of vertical humidity gradients. Testing these findings via machine learning (Gradient Boosting, LightGBM, Random Forest) showed that incorporating lidar-derived vertical features reduces PM_2.5_ prediction error. While MAIAC AOD remains a critical predictor, model performance degrades in mixed-layer regimes compared with deep-dust environments. We conclude that reliable satellite-based monitoring requires transitioning from bulk column-integrated assumptions to frameworks incorporating vertical aerosol layering and atmospheric residence time.

## Graphical abstract

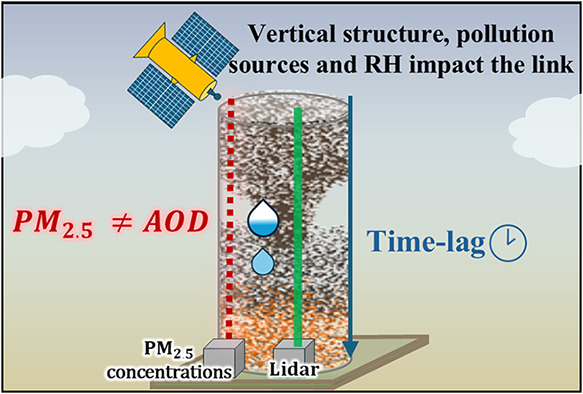

## 1 Introduction

Monitoring fine particulate matter (PM_2.5_) is essential for epidemiological studies, yet limitations in the spatial density of ground-based monitoring networks demand the use of alternative observations.^1^ Satellite remote sensing of aerosol optical depth (AOD) has emerged as a primary tool for bridging this observational gap, providing the global spatiotemporal coverage.^2–5^ Polar^6–8^ and geostationary^9,10^ satellite instruments provide AOD product for estimating PM_2.5_ from space. Combining AOD with meteorological and additional environmental data in machine learning (ML) algorithms enhances the representation of nonlinear relationships between satellite-derived observations and ground-level air pollution.^11–19^ However, the utility of AOD as a proxy for surface air quality relies on the geophysical relationship between the column-integrated optical signal and mass concentration at the surface.^20^ This relationship is neither static nor linear; it is modulated by aerosol composition, boundary layer, hygroscopic growth, and, most critically, the vertical distribution of the aerosol load.^9,21–27^ Despite these well-recognized limitations, satellite observations remain central to PM_2.5_ estimation because no alternative observing system provides comparable spatial coverage, consistency, and long-term measurements.

Most studies modeling satellite-retrieved AOD with surface PM_2.5_ treat AOD as a single column-integrated value, overlooking its variable vertical structure. When vertical information is included, it typically comes from four sources: (i) space-borne lidar extinction profiles (primarily CALIPSO/CALIOP), (ii) ground-based ceilometer/lidar estimates of mixing-layer/boundary-layer height (BLH), (iii) in situ aircraft profiles from intensive campaigns, and (iv) model-simulated vertical structure (e.g., GEOS-Chem) used to translate column AOD to near-surface mass.^28–331^ These efforts, largely conducted in East Asia,^28,31,32^ North America,^33,34^ and Europe,^35^ have consistently shown that vertical context is important. In the Eastern Mediterranean (EM), studies by Kloog et al.^36^ and Shtein et al.^37^ modeled PM_2.5_ over Israel, but without accounting for vertical layering. Case studies of lofted smoke illustrate the limitation: high AOD can reflect free-tropospheric aerosol while surface PM_2.5_ remains low, an ambiguity resolved only with vertical constraints.^28^ Still, most work reduces profiles to broad categories such as dust vs nondust, missing the complexity of real multilayered structures.^38^ Figure 1b highlights the challenge of the low correspondence between satellite-based AOD and surface-level PM_2.5_.

**Figure 1:**
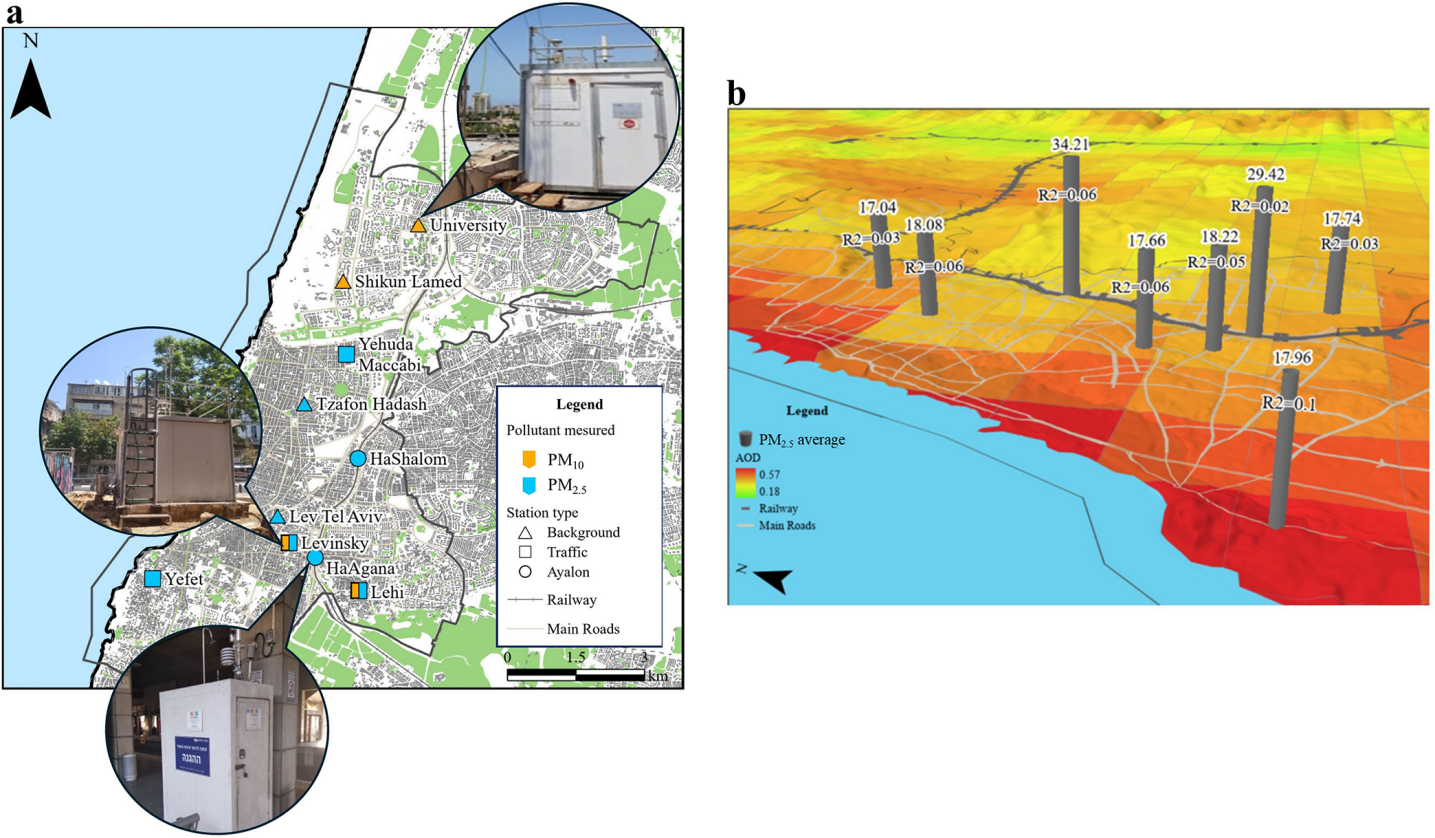
Panel (a): Map of the study area. Environmental monitoring stations are represented by different shapes according to their classification: triangles, background stations; squares, traffic stations; and circles, Ayalon stations. Colors indicate the measured pollutant type: blue for PM_10_ and green for PM_2.5_. Panel (b): 3D map of the study area. Environmental monitoring stations are represented as vertical columns, where the height of each column indicates the average PM_2.5_ concentration during the study period (value shown above each column). AOD is displayed as a pixel-based background layer. The coefficient of determination (*R* ^2^) between AOD and PM_2.5_ is indicated in each column. Note the very low *R* ^2^ values that vary between 0.02 and 0.1.

Our recent work established a classification of vertical aerosol layering conditions in the EM and demonstrated its impact on MAIAC (Multi-Angle Implementation of Atmospheric Correction) AOD retrieval accuracy.^26^ However, how these vertically resolved aerosol structures translate into ground-level particulate mass concentrations (PM_2.5_) remains unclear. Many satellite-based PM_2.5_ approaches use land use and meteorological descriptions of ground monitoring stations, assuming a vertically homogeneous aerosol column,^36^ neglecting layered air-mass configurations that can decouple column AOD from surface exposure. Building on our classification of 10 aerosol vertical structure types, ranging from single-layer to complex multilayer regimes, this study examines how vertical aerosol structure systematically modulates the AOD–PM_2.5_ relationship. To our knowledge, this is the first analysis to address this link using a stratified, physically based framework.

### 1.1 Novelty of the Study

- Conditioning surface ground particulate pollution on vertical aerosol layering: We investigate whether the AOD-PM_2.5_ relationship is strictly layering-dependent. By analyzing each layering type separately, we identify the configurations in which AOD remains a reliable proxy for PM_2.5_ and those in which the satellite signal becomes nonrepresentative.
- Integrating temporal and settling dynamics: We introduce a spatiotemporal sensitivity matrix-testing variable, spatial buffers, and temporal lags (including previous-day signals) to determine if accounting for turbulent downward mixing and atmospheric residence time improves the link.
- Incorporating altitude-specific relative humidity (RH) (lidar-based) profiling (at ground, 1000 m, and 3000 m): We aim to determine how humidity-dependent particle growth distorts the satellite AOD signal relative to surface PM_2.5_.
- Evaluating vertical dependencies on the AOD-PM_2.5_ relationship across street-level and rooftop monitoring stations: We aim to better understand the satellite’s sensitivity to regional aerosol transport layers versus its ability to resolve local, near-surface anthropogenic emissions.

## 2 Study Area and Classification of the PM_2.5_ Monitoring Stations

Tel Aviv metropolitan is located on the Mediterranean coast of central Israel and is one of the country’s most densely populated areas. Tel Aviv experiences significant anthropogenic activity, particularly related to traffic and urban development. To monitor air quality, Tel Aviv is equipped with a network of ground-based environmental monitoring stations that continuously measure concentrations of fine particulate matter (PM_2.5_). These stations are part of the national environmental monitoring network.

The monitoring stations are categorized based on their location and the surrounding environment. ‘Background’ stations follow the official classification by the Ministry of Environmental Protection and are positioned on rooftops to reduce the impact of local emissions. While they are intended to represent urban background conditions, they are located within the city and may still be influenced by diffuse urban emissions. “Traffic” stations are located near busy streets to capture primarily vehicle-related pollution. Within the traffic category, we further distinguish a subset of stations located along the Ayalon Highway: a major transportation corridor running through Tel Aviv. These “Ayalon” stations are situated within train stations along the highway. As such, they are considered semi-indoor environments and reflect unique pollution characteristics influenced by both road traffic and nearby railway activity.

Figure 1a shows the spatial distributions of the monitoring stations. A photograph of an example station is provided for each category to illustrate typical location conditions.

## 3 Methodology

Briefly, we first categorized the ground stations measuring PM_2.5_ into three groups: (1) background stations, (2) traffic stations, and (3) Ayalon highway stations. We then analyzed the average PM_2.5_ concentration for each station and for each station type. Due to very low *R* ^2^ between AOD vs PM_2.5_,^39^ as presented in Figure 1b, we investigated the *R* ^2^ between PM_2.5_ and AOD under various conditions, considering different pollution layering types as defined in Rogozovsky et al.^26^ Finally, we examined the ability of three (Random Forest, Gradient Boosting, and Light Gradient-Boosting Machine) machine learning (ML) algorithms to predict PM_2.5_ concentrations under different layering conditions.

### 3.1 Data

#### 3.1.1 PM_2.5_ Measurement and Thresholds Guidelines Criteria

Long-term PM_2.5_ trends (2000–2024) are analyzed separately from the vertically resolved satellite–lidar analyses (2019–2024). Hourly PM_2.5_ measurements were obtained from Israel’s national environmental monitoring network. We analyzed data from 2000 to 2024 to describe concentration levels across different station types and compared them with air quality standards. For consistent interstation comparison, analyses were limited to days when all stations (*N* = 8) reported valid data, resulting in 890 shared days. Daily mean concentrations and MAIAC-matched averages for 09:00–13:00 local time were calculated and used for association, trend, and predictive analyses. Integration with lidar-derived vertical profiles and MAIAC aerosol optical depth (AOD) data was available only for 2019–2024. The 2021 World Health Organization (WHO) Global Air Quality Guidelines recommend a 24-h PM_2.5_ limit of 15 μg/m^3^,^40^ which matches Israel’s national “Target” value.^41^ Israel’s Clean Air Law also defines a legally binding “Environmental” standard of 37.5 μg/m^3^; concentrations above this level constitute a regulatory violation.^41^

PM_2.5_ concentrations are measured at monitoring stations operated by the Ministry of Environmental Protection using continuous monitoring techniques, primarily Tapered Element Oscillating Microbalance (TEOM)^42^ and β Attenuation Monitors (BAM).^43^ TEOM instruments measure particle mass by collecting aerosols on a vibrating filter element, while BAM instruments determine particle mass through the attenuation of β radiation as particles accumulate on filter tape.

#### 3.1.2 MODIS-MAIAC AOD Retrieval

The MAIAC algorithm was used to retrieve daily AOD values from the Moderate Resolution Imaging Spectroradiometer (MODIS) aboard the Terra and Aqua satellites.^44^ The MAIAC product provides AOD at a 1 km spatial resolution, corrected for cloud and surface effects, and a part of conventional AOD product (MCD19A2: MODIS/Terra + Aqua Land Aerosol Optical Depth Daily L2G Global 1 km SIN Grid). For each PM_2.5_ station, we extracted the AOD of the closest pixel, and a 3 × 3 pixel average around each station. MAIAC AOD was used both as a predictor in ML models and for assessing temporal trends and biases under various aerosol layering conditions.

#### 3.1.3 Aerosol Robotic Network (AERONET) Measurements

AERONET is a ground-based sun photometer network that provides high-accuracy measurements of aerosol optical properties. In this study, data were primarily obtained from the Tel Aviv University AERONET site. Data gaps were filled using observations from the Weizmann Institute AERONET site, situated about 27 km from Tel Aviv University. The resulting coefficient of determination (*R* ^2^ = 0.93) demonstrates strong consistency between the two data sets.^45^ We used AOD measurements at multiple wavelengths and interpolated them to 470 nm for consistency with the MAIAC AOD. In addition, the Ångström exponent at 440–675 nm was used to estimate aerosol particle size, enabling the distinction between coarse-mode aerosols (e.g., dust) and fine-mode aerosols (e.g., anthropogenic pollution).^39,46^

#### 3.1.4 PollyXT-Lidar

Vertical aerosol profiles were obtained using the PollyXT multiwavelength Raman lidar system operated at Tel Aviv University.^47^ The lidar provides range-resolved backscatter and extinction profiles at multiple wavelengths, allowing detailed characterization of the aerosol layer height, depth, structure, and number of aerosol layers. This information was used to examine how vertical structure affects both MAIAC AOD accuracy and the AOD–PM_2.5_ relationship.

Aerosol optical properties derived from lidar observations include the following parameters: the particle extinction coefficient (α), which quantifies the attenuation of radiant energy by scattering and absorption; and the particle backscatter coefficient (β), which indicates the strength of the lidar return signal and typically increases under polluted or dust-laden conditions. The particle lidar ratio (LR), defined as α_λ_/β_λ_, varies with aerosol type, with low values (<30 sr) characteristic of marine aerosols, higher values (50–80 sr) of anthropogenic pollution, and intermediate values (35–40 sr) typical of dust.^48^ The particle depolarization ratio (δ_p_) differentiates spherical from nonspherical particles based on the ratio of perpendicular to parallel backscatter; values close to zero indicate spherical fine-mode pollution, while higher values (∼0.39) denote coarse, nonspherical dust.^49^ Together, these parameters provide a detailed characterization of the aerosol type and loading in the atmosphere.

#### 3.1.5 Vertical Aerosol Layering Conditions

We used the previously identified 10 distinct vertical aerosol layering conditions (A–J) in the EM, based on the lidar target classification product.^50^ These conditions represent a range of vertical structures and aerosol mixing states, from purely anthropogenic layers to complex multilayered mixtures in which marine aerosols, anthropogenic pollution, and dust occupy different altitudes. This classification captures systematic differences in aerosol type, vertical distribution, and meteorological parameters.^26^ A schematic legend of the layering conditions is presented in Figure 3, panel a. To merge PM_2.5_ measurements with column-integrated AOD, we applied the vertical layering classification (types A–J) from Rogozovsky et al.^26^ Following the surrogate-measurement logic of Sarnat et al.,^51^ atmospheric layering can serve as a proxy for aerosol composition, allowing analysis of AOD-PM_2.5_ relationships within distinct atmospheric states.

#### 3.1.6 Relative Humidity (RH) Data

The RH is obtained by combining the Raman lidar observation of the water-vapor-to-dry air mixing ratio (WMR) with modeled temperature information.^52^ The uncertainty is of the order of 10–15%.^52,53^

To validate the lidar-derived RH, we compared it with radiosonde noon and midnight measurements from the Beit Dagan meteorological station. The radiosonde profiles were used to assess the consistency of the lidar-based RH retrievals (Figure S1).

We studied RH under different aerosol layering conditions (A–J) across three height ranges: (1) ground-level RH, (2) layer-averaged RH between 0 and 1000 m, and (3) layer-averaged RH between 1000 and 3000 m. Additionally, the RH values from height ranges 2 and 3 were used as inputs in the PM_2.5_ prediction models (Section 3.3).

Humidity strongly influences aerosol optical properties through hygroscopic growth. To account for this, we used lidar-retrieved vertical RH profiles as a proxy for composition-driven optical enhancement. Previous studies show that aerosol composition and vertical structure significantly affect the AOD-PM_2.5_ relationship and that water uptake can substantially increase AOD under humid conditions.^25,54,55^ PM_2.5_ concentrations are not routinely provided, so only total PM_2.5_ mass was used. However, RH-segregated lidar profiles still capture key differences between the dust and pollution regimes.

### 3.2 Assessing the Explanatory Power of AOD for Ground-Level PM_2.5_ Estimation

We used environmental monitoring stations located in Tel Aviv, which include three types: traffic, background, and Ayalon highway stations (Section 2). The average daily PM_2.5_ concentrations at each monitoring station were compared to both Israeli and WHO guidelines criteria.^40^ Next, we examined the temporal variation in annual PM_2.5_ concentrations for background and traffic stations only. Ayalon highway stations were excluded from this analysis, as their measurements are strongly influenced by highway and train traffic.

Next, we analyzed the *R* ^2^ between MAIAC AOD and PM_2.5_ concentrations under different aerosol layering conditions. We used the following configurations: (1) For PM_2.5_, we used both the daily average and the average between 09:00 and 13:00, corresponding to the MAIAC overpass time. (2) For AOD, we examined four variations: the average of a 3 × 3 pixel window surrounding each PM_2.5_ monitoring station, a single pixel located directly over each station, and the same two spatial approaches but with a one-day temporal shift (i.e., referring to AOD values recorded 1 day before the PM_2.5_ data). This analysis was conducted separately for background and traffic stations to account for differences in emission sources and local atmospheric conditions.

### 3.3 Machine Learning for PM_2.5_ Estimation: Studying the Impact of Layering Conditions

To evaluate the contribution of the lidar variables to PM_2.5_ estimation, each model was trained twice: including the lidar variables (MAIAC + AER + MET + LIDAR) and excluding them (MAIAC + AER + MET). For the estimation of surface PM_2.5_ concentrations, we implemented and compared three ML algorithms: Gradient Boosting (GB),^56^ Light Gradient-Boosting Machine (LightGBM),^57^ and Random Forest (RF).^58^ These specific ensemble-based learners were selected for their proven capacity to handle the nonlinearities and complex interactions typical of atmospheric data sets, particularly when integrating satellite-derived AOD with heterogeneous meteorological variables. While GB and LightGBM offer high predictive accuracy through their iterative error-reduction (boosting) approach, RF was included to provide a robust baseline that is less prone to overfitting and better suited for identifying the hierarchical importance of diverse features.^59–61^

To optimize model performance, a feature importance analysis based on Mean Decrease in Impurity^62^ was conducted to identify the most influential predictors. This procedure allowed us to isolate variables that contributed most significantly to the PM_2.5_ predictions. The top-ranking variables were used as inputs for the final models presented in Table 1.

**Table 1:** Overview of Variables Used in This Study and Their Data Sources

source	feature	details
AERONET (AER)	Ångström exponent_440–675 nm_	Indicates aerosol particle size
University ground station (MET)	Air Temperature	Represents near-surface air temperature at 2m height
	Wind speed	Controls aerosol transport and dispersion
	Wind direction	Indicates dominant air-mass origin
MODIS-MAIAC	AOD_470 nm_	Quantifies the column-integrated aerosol optical loading
Lidar	RH 0–1000 m	Characterizes moisture conditions within the planetary boundary layer
	RH 1000–3000 m	Characterizes moisture conditions in the lower free troposphere
	LR_532nm_ 1000–3000 m	Provides information on aerosol type
	δ_*p*532nm_ 0–1000 m	Differentiates spherical from nonspherical particles within the boundary layer
	δ_*p*532nm_ 1000–3000 m	Differentiates spherical from nonspherical particles in the lower free troposphere

Each model was assessed using 10-fold cross-validation, where the data set was divided into 10 subsets. In each iteration, one subset was used for testing, while the remaining nine were used for training. This process was repeated 10 times. During each iteration, the models were evaluated using several performance metrics, including the *R* ^2^, root mean squared error (RMSE), and mean absolute error (MAE). Finally, the average values of each metric across all 10 iterations were calculated.

Finally, we run the models on groups divided according to the AOD–PM_2.5_ relationship-focused model (Section 3.2). Each group contained 2–3 layering types, classified based on the AOD–PM_2.5_ correspondence and MAIAC reliability, allowing us to evaluate if model performance varies across different layering regimes.

## 4 Results

### 4.1 PM_2.5_ Daily Concentrations

Figure 2 illustrates daily PM_2.5_ concentrations across the monitoring stations, categorized by station type (orange: Ayalon highway; green: urban traffic; purple: background). The “Target” (15 μg/m^3^) and “Environmental” (37.5 μg/m^3^) guidelines, as established in the previous section, are marked by red dashed lines for quantitative comparison. As can be seen, the average PM_2.5_ concentration at Ayalon stations is 31.83 μg/m^3^, which is approximately 56% higher than the average concentrations observed at traffic or background stations. As demonstrated by the elevated values, these stations capture highly localized emissions rather than represent the broader urban environment. Consequently, their PM_2.5_ measurements are not expected to correspond well with satellite-derived AOD values. Therefore, in the following analyses, we focus solely on traffic and background stations. Note that there is an overlap in the distributions of PM_2.5_ at the traffic and nontraffic urban stations. However, the traffic sites consistently exhibit higher median and upper quartile concentrations, reflecting the expected influence of nearby road emissions, even within the densely mixed Tel Aviv urban environment.

**Figure 2:**
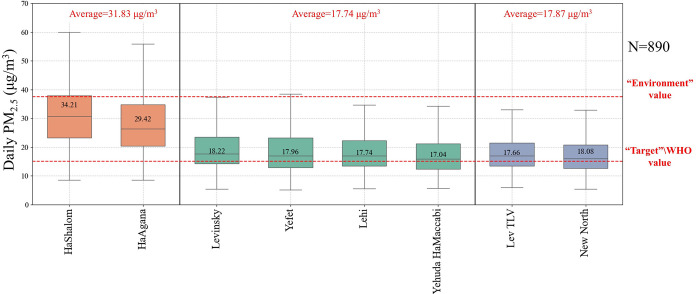
Boxplot of PM_2.5_ concentrations measured at ground stations in Tel Aviv. The stations are categorized into three groups: Ayalon (orange), traffic (green) and background (purple). The average PM_2.5_ concentration for each group is indicated above the corresponding group of boxplots. The daily target and environmental threshold values are marked with red dashed lines.

### 4.2 Explanatory Power of AOD for Ground-Level PM_2.5_ Estimation

#### 4.2.1 Vertical Layering Conditions Impact

Figure 3, panel a, shows the *R* ^2^ (gray scale) between the MAIAC AOD retrieval and the ground AERONET AOD, conditioned on the aerosol layering types (A–J)^26^ (illustrated schematically). Figure 3, panel b, shows the heat map of the relationship between AOD and PM_2.5_ concentrations. These relationships are shown for each layering type (A–J). The color scale on a heat map indicates the statistical agreement strength, ranging from white/light (low) to dark red (high). As we noted above, for the analyses we used two types of measurement sites (background and traffic) and two temporal averaging periods (daily and 9:00–13:00). Additionally, we examined different spatial aggregations, where group a (a1–a4) represent 3 × 3 pixel average around each station and group b (b1–b4) is for one AOD pixel for each station.

**Figure 3:**
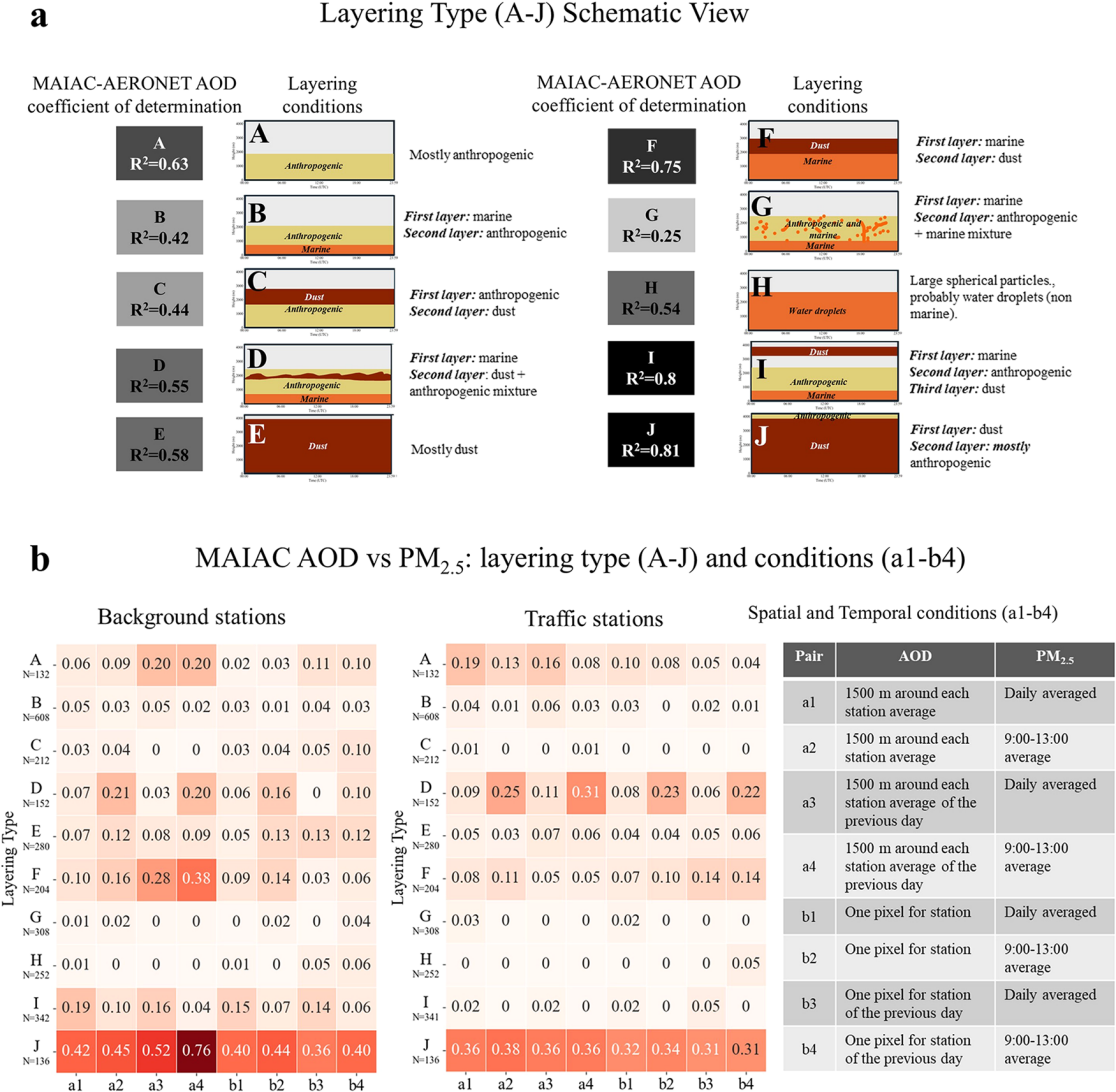
Panel (a): A schematic legend for the aerosol layering types A–J, as defined in Rogozovsky et al.^26^ For each layering type, the *R* ^2^ between AERONET and MAIAC AOD is presented in gray scale colors (dark, high; light, low). Panel (b): Heatmaps showing the *R* ^2^ between PM_2.5_ and AOD for the background (left) and traffic stations (right). Eight different configurations of PM_2.5_ versus AOD are described in the accompanying table.

Table 2 outlines the classification of aerosol vertical layering types into operational reliability categories based on both the MAIAC retrieval performance (Figure 3, panel a) and the strength of the AOD–PM_2.5_ relationship (Figure 3, panel b). As can be seen, high optical retrieval accuracy does not imply surface representativeness when aerosol layers are vertically decoupled. Generally, as evident from our analyses, the *R* ^2^ between satellite AOD and surface PM_2.5_ exhibits a strong dependence on the aerosol vertical structure (panel b). The most robust relationships were found under the regime characterized by a deep-dust layer with an anthropogenic layer above (J), consistently yielding the highest correspondence (≈0.36–0.76). This result is not surprising, as MAIAC algorithm divides the globe into regional tiles and, for the EM, predominantly applies a predefined dust aerosol model optimized for deep, coarse-mode dust layers with limited anthropogenic influence.^44^

**Table 2:** Classification of Aerosol Vertical Layering Types into Operational Reliability Groups^a^

reliability	layering types	MAIAC retrieval accuracy	AOD-PM_2.5_ relationship	dominant physical mechanism	operational recommendation
Coherent	F, J	High (*R* ^2^ > 0.8)	High agreement	Stratified layers where column loading is proportional to surface mass	High confidence: Suitable for operational monitoring; lag-time corrections improve accuracy
Spatial–temporal dependency	A, E	Moderate (*R* ^2^ ≈ 0.58–0.63)	Depend on the spatial/temporal scenario	Homogeneous layer (anthropogenic or advected dust layer)	Conditional use: Requires considering spatial and temporal variations
Misleading	D, I	Moderate-High (*R* ^2^ ≈ 0.55–0.8)	Low agreement	Elevated aerosol layers do not mix downward into the surface boundary layer	Caution: High optical accuracy, low surface relevance
Unreliable	B, C, G	Low (*R* ^2^ < 0.45)	No agreement	Mixed marine and anthropogenic, or anthropogenic with upper dust layer aerosols overwhelm the retrieval algorithm	Data rejection: Retrievals in this regime are physically unreliable

a The categorization integrates the intrinsic MAIAC retrieval accuracy (AERONET—MAIAC AOD correspondence, as presented in Figure 3a) and the strength of the surface AOD-PM_2.5_ coupling (Figure 3 b).

Conversely, the agreement between both parameters approached zero in both the highly mixed, low-accuracy type G and the shallow type A anthropogenic layers. Importantly, despite Type A maintaining moderate MAIAC accuracy (0.63), the resulting AOD-PM_2.5_ agreement remained low (≈0.06–0.20). This outcome may indicate that in shallow layers, the total column AOD is insensitive to fluctuations in ground-level PM_2.5_ concentration.

#### 4.2.2 Site Location

Our results indicate that the location of the observation point and nature of the pollution source are also critical factors in this complex AOD vs PM_2.5_ relationship. In particular, background stations yield stronger AOD-PM_2.5_ agreement across most layering types and configurations (especially for a1–a4) than traffic sites. This suggests that the high variability and localized nature of traffic-related emissions at the surface disrupt the relationship with larger-scale, columnar AOD measurements. The spatial averaging effect of background stations makes satellite-derived AOD more representative of PM_2.5_.

#### 4.2.3 Temporal and Spatial Averaging

Our results suggest that short-term temporal averaging of PM_2.5_ (that aligns with MAIAC overpass time, 9–13 UTC), which also involves previous-day AOD averaging, enhances agreement. Combinations a4 and a3 (previous-day AOD) produce higher *R* ^2^ values in several layering types (especially types J and F in Background stations). This result may indicate a lag effect or temporal smoothing that enhances satellite-ground agreement during dust conditions (as dust settles over time). Similarly, MAIAC-time PM_2.5_ averaging (9:00–13:00) appears to improve *R* ^2^ in some cases (e.g., *a*4 > *a*3 in type J).

There are different statistical agreements for daily and 9:00–13:00 averages that hint at the impact of the diurnal cycle of air pollution. The 9:00–13:00 window is a period of rapid PBL growth and vertical mixing. The high agreement for type J during this specific window suggests that this mixing process may be what aligns the columnar AOD with the surface PM_2.5_ concentration under these particular layering conditions. The daily average, which includes periods of a shallow morning PBL and a collapsed evening PBL, would average out these dynamics.

Pixel-level AOD (b1–b4) performs worse than averaged AOD (a1–a4) across both station types. In particular, it shows lower *R* ^2^ values compared to spatially averaged MAIAC AOD retrievals (a1–a4), likely due to the impact of the background surface reflectance on the accuracy of MAIAC AOD retrievals, as shown by Sever et al.^63,64^ (Table 1S in Sever et al.^63^).

#### 4.2.4 Changes in RH for Different Layering Conditions

Figure 4 illustrates the vertical distribution of RH at ground level, 0–1000 m, and 1000–3000 m across the identified layering types. These profiles provide the physical context needed to interpret the variation in the AOD-PM_2.5_ relationship. A distinct feature of our data set is the persistently high RH observed in the lowest atmospheric layers. Reflecting Tel Aviv metropolitan coastal location influenced by the marine boundary layer, rather than a desert environment, ground-level RH consistently exceeds 70% for the majority of layering conditions (types A–D, F–I). Importantly, this moisture is not confined to the surface; the 0–1000 m measurements indicate that humid conditions (RH ≈ 60–70%) extend through the lower boundary layer. Although the lidar RH profiles are retrieved postsunset, the values at 0–1000 m are considered representative of the daily average conditions governing the aerosol column as represented by the radiosonde (Figure S1).

**Figure 4:**
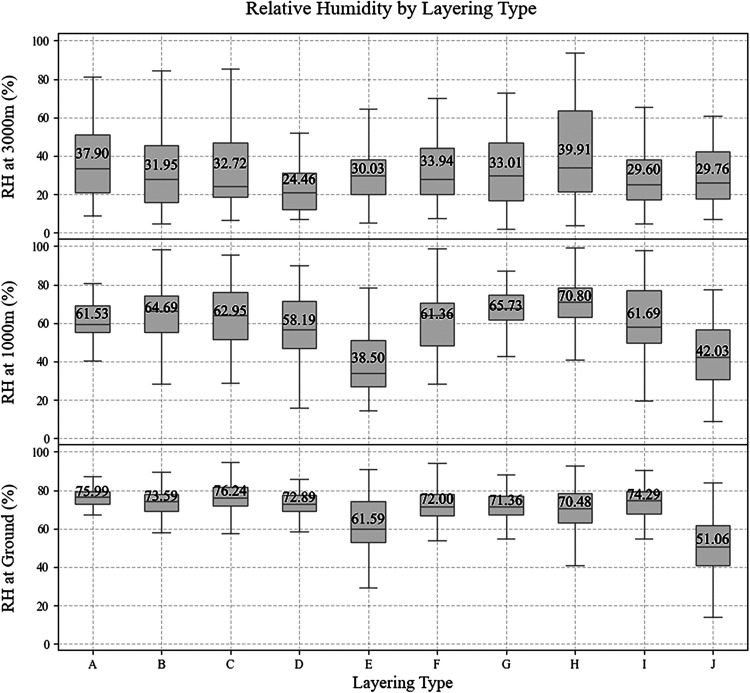
Boxplots of relative humidity (RH) at ground level (lower plot) measured by the environmental station “University” in Tel Aviv. The RH at 1000 m and 3000 m altitude is measured by lidar. Each color represents the layering type (A–J). The text on each box indicates the mean value.

Our results hint that there is a “hygroscopic growth vs. dry mass conditions” mechanism.^65,66^ The atmospheric layer from the surface to 1000 m contributes the largest fraction to the total MAIAC AOD. Within this humid layer, hygroscopic aerosols (such as marine salts and urban sulfates) undergo water uptake.^67,68^ This hygroscopic growth increases the particle scattering cross section, increasing the AOD value without a corresponding increase in PM_2.5_. Conversely, hydrophobic particles, such as mineral dust, remain unaffected by these variations in water vapor. As a result, in these “humid” regimes, the variable rate of water uptake introduces a complex nonlinearity between the columnar AOD signal and PM_2.5_. Similar sensitivities of the AOD–PM_2.5_ relationship to vertical humidity structure and atmospheric dynamics have been reported over tropical environments.^69^

As shown in Figure 4, type J is the distinct outlier, characterized by a “dry” regime with the lowest mean RH at both the surface (51.06%) and 0–1000 m (42.03%). In this dry column, hygroscopic growth is minimized, allowing the optical properties of the aerosols to linearly better reflect their dry mass concentrations, yielding the most robust AOD-PM_2.5_ *R* ^2^ (Figure 3).^52,53,70^

In cases where marine aerosols dominate the surface layer (layering types D–I), background stations exhibit higher *R* ^2^ values (Figure 3). These particles remain consistently swollen with water (RH ∼ 80%) and rarely dry out, such that variations in RH cause minimal influence, similarly to hydrophobic dust layers.

### 4.3 Estimation of PM_2.5_: Insights into the Model Development

To evaluate the contribution of vertical profile data to surface PM_2.5_ estimation, we compared the model performance with and without lidar-derived covariates. Table 3 presents the results of 10-fold cross-validation (Section 3.3) for the entire study period using three machine learning algorithms: GB, LightGBM, and RF. As can be seen, incorporating vertical parameters (lidar ratio, depolarization ratio δ_*p*_, and altitude-specific RH) yielded a consistent improvement in the predictive power across all studied models. The LightGBM model integrating lidar inputs achieved robust generalization, increasing the testing *R* ^2^ from 0.61 to 0.65 and reducing the RMSE from 5.78 to 5.43 μg/m^3^. This reduction in residual variance confirms that standard meteorological and columnar optical variables are insufficient to fully capture surface heterogeneity. The lidar parameters constrain the vertical allocation of the aerosol column, correcting for measurements where optical load does not translate to surface mass. It should be noted that because lidar measurements originate from a single site, they do not contribute station-specific spatial variability, explaining the modest *R* ^2^ improvement in the ML models despite the strong regime-dependent differences revealed by the lidar-based A–J classification in our correlation analyses.

**Table 3:** Cross-Validation Results for the Gradient Boosting, Random Forest, and LightGBM Models, Evaluated under Two Variable Configurations^a^

model	variables	*N*	*R* ^2^ train.	*R* ^2^ test	RMSE train.	RMSE test	MAE train.	MAE test
Gradient Boosting	(1) MAIAC + AER + MET + LIDAR	2453	0.81	0.63	4.04	5.60	2.93	3.91
Gradient Boosting	(2) MAIAC + AER + MET		0.79	0.60	4.34	5.87	3.20	4.15
Random Forest	(1) MAIAC + AER + MET + LIDAR	2453	0.76	0.62	4.55	5.68	3.27	4.03
Random Forest	(2) MAIAC + AER + MET		0.73	0.59	4.85	5.95	3.53	4.26
LightGBM	(1) MAIAC + AER + MET + LIDAR	2453	0.82	0.65	4.03	5.43	2.76	3.72
LightGBM	(2) MAIAC + AER + MET		0.77	0.61	4.50	5.78	3.18	4.06

a (1) All variables (AERONET angstrom exponent at 440-675 nm, MAIAC AOD 470 nm, ground measured temperature, wind speed and wind direction, and the next lidar parameters: RH at 0–1000 m and 1000–3000 m, lidar ratio 532 nm at 1000–3000 m, particle depolarization ratio 532 nm at 0–1000 m and 1000–3000 m) included (2) excluding lidar variables. The table presents *R* ^2^, root mean square error (RMSE), and mean absolute error (MAE).

To identify the primary drivers of PM_2.5_ variability under different layering regimes, we examined the feature importance scores from the models used in this study (Figure 5). In the MAIAC + AER + MET + LIDAR model, wind speed was the top-ranked predictor for both RF and GB, whereas for LightGBM, MAIAC-derived AOD was the most important variable. For the other models, the AOD ranked second in importance. Wind speed is a key predictor of PM_2.5_ as it governs aerosol transport, dispersion, and near-surface concentrations (low wind speeds lead to the accumulation of pollution near the surface^71^). The high importance of AOD highlights the value of satellite-based column measurements for estimating surface PM_2.5_, particularly under complex or variable vertical profiles. Notably, MAIAC AOD provides spatially unique values for each PM_2.5_ monitoring station, extracted from the corresponding satellite pixels each day.^72^ While all other features (lidar, AERONET, and meteorological data) also vary from day to day, they are obtained from a single location, not colocated with the individual monitoring stations. Therefore, for each day, MAIAC AOD varies for each monitoring station, reflecting local conditions, whereas all other features represent the broader regional environment.

**Figure 5:**
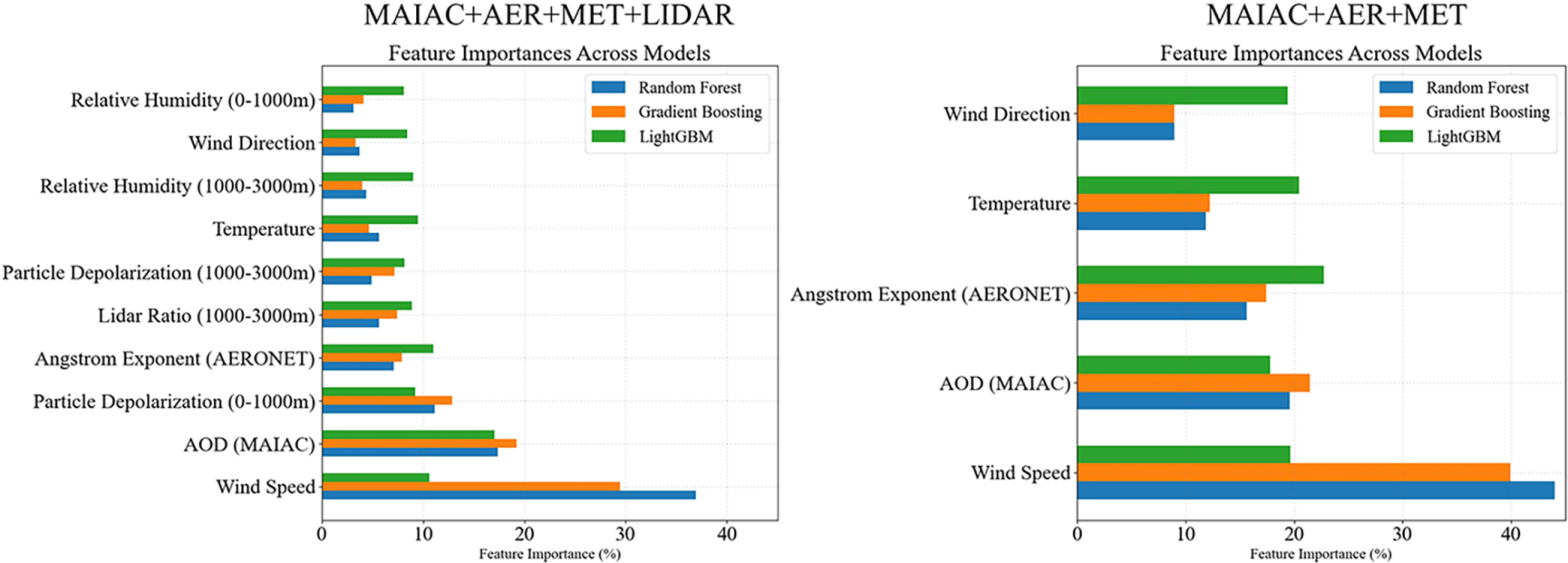
Feature importance for the RF (blue), GB (orange), and LightGBM (green) models predicting PM_2.5_ concentrations. The figure on the left features importance for the models, including lidar data (MAIAC + AER + MET + LIDAR) and the one on the right models excluding lidar data (MAIAC + AER + MET).

The implementation of a regime-based modeling framework, stratifying the data based on operational reliability groups (as classified in Table 2), validated the central hypothesis that neglecting the vertical aspect of the atmosphere can be crucial. The models presented in Table 3, which account for all layering types together, yielded a global cross-validated *R* ^2^ of 0.59–0.63 and RMSE of 5.43–5.95 μg/m^3^. In contrast, regime-stratified models (Table 4) exhibited varying predictive performance, corresponding to the specific physical processes within each layer. The models trained for the “Coherent” (layer types J and F) and “spatial–temporal dependent” (A, E) structures demonstrated robust predictive power. The success of these models ensures high-quality estimates during periods of elevated aerosol concentrations, which correlate most strongly with acute public health risks. Conversely, the models for the “Misleading” (D, I) and “Unreliable” (B, C, G) regimes returned low predictive ability. This poor performance, perhaps, can be used as a diagnostic filter: it confirms that during these states, the satellite-derived column AOD is either physically separated from ground PM_2.5_ or optically disturbed by complex mixtures.

**Table 4:** Cross-Validation Results for the Gradient Boosting, Random Forest, and LightGBM Models, Grouped by Operational Reliability Groups as Classified in Table 2 ^a^

model	variables	N	*R* ^2^ train.	*R* ^2^ test	RMSE train.	RMSE test	MAE train.	MAE test
Gradient Boosting	F, J	249	0.99	0.67	1.57	7.33	1.21	5.35
Gradient Boosting	A, E	277	0.98	0.74	1.47	5.08	1.11	3.80
Gradient Boosting	D, I	304	0.95	0.21	1.19	4.19	0.91	3.12
Gradient Boosting	B, C, G	785	0.86	0.49	2.23	4.18	1.66	2.98
Random Forest	F, J	249	0.83	0.66	5.52	7.42	3.86	5.31
Random Forest	A, E	277	0.87	0.74	3.79	5.15	2.79	3.87
Random Forest	D, I	304	0.70	0.32	2.94	3.92	2.13	2.96
Random Forest	B, C, G	785	0.72	0.53	3.18	4.03	2.23	2.92
LightGBM	F, J	249	0.88	0.70	4.66	6.95	3.28	4.96
LightGBM	A, E	277	0.92	0.77	3.00	4.76	2.22	3.55
LightGBM	D, I	304	0.78	0.35	2.53	3.83	1.77	2.86
LightGBM	B, C, G	785	0.80	0.53	2.71	4.00	1.87	2.82

a The table presents *R* ^2^, root mean squared error (RMSE), and mean absolute error (MAE).

## 5 Discussion and Future Directions

This study investigated the relationship between ground-level PM_2.5_ concentrations and satellite-derived AOD in the Tel Aviv metropolitan area by integrating surface monitoring, vertical lidar variables, and machine learning models. Generally, PM_2.5_ concentrations declined from 30 μg/m^3^ in 2000 to 17 μg/m^3^ in 2024, while AOD remained stable (0.25–0.30; Figure S2). The decrease in PM_2.5_ is consistent with regulatory changes, particularly the 2008 implementation of Israel’s Clean Air Law^41^ and its revision in 2021 to align with updated WHO guidelines.^40^ These policies likely played a key role in reducing the urban particulate levels.

The statistical agreement between AOD and PM_2.5_ was found to be highly dependent on the vertical structure of the atmosphere.^23,25,73^ Layering type J consistently generated the highest *R* ^2^ values across station types and different pairing methods, indicating that under this condition, satellite-derived AOD provides a reliable proxy for surface PM_2.5_. This is not surprising, as previous studies have shown that under Middle Eastern dust conditions (corresponding to layering type J), the MAIAC algorithm performs best,^26^ as it fits the MAIAC model assumptions for this region.^44^

At first glance, layering type E appears structurally similar to type J, which could suggest comparable AOD-PM_2.5_ behavior. However, type E reflects North African dust, while type J corresponds to Middle Eastern dust,^26^ and the two differ in terms of mineralogy and particle size distribution.^74,75^ In particular, the North African dust storm has more sodium chloride^76^ and organic particles, while the Middle Eastern storm is rich in palygorskite mineral and differ quantitatively in such minerals as Illite–smectite, kaolinite, and iron oxides, as shown in Figure 3 and Table 1 of Ganor et al.^77^ These differences alter the optical properties of the layer,^78^ reducing the AOD–PM_2.5_ correspondence for type E. Thus, despite their superficial similarity, type E produces lower *R* ^2^ values, highlighting the role of dust source and mineralogical composition in shaping the AOD–PM_2.5_ relationship.

Layering types A–H showed weak or no agreement between satellite and ground measurements, suggesting that in these scenarios, the MAIAC algorithm struggles to correctly calculate the AOD, reducing the sensitivity of AOD to ground-level concentrations.^23^ Background stations exhibited stronger AOD–PM_2.5_ correspondence than traffic stations, likely due to the greater representativeness of the surrounding environment. Additionally, we found that comparing PM_2.5_ concentrations with AOD values from the previous day resulted in improved correspondence, likely due to lofted dust layers that mix down to the surface.^79^

The discrepancy between dry mass concentrations measured at the monitoring station and satellite-derived AOD may, in part, reflect the loss of semivolatile species, such as ammonium nitrate and secondary organic aerosols.^80–82^ Sarnat et al.^51^ demonstrated that pollutant correlations are highly dependent on the atmospheric state. Accordingly, we classify profiles into layering types A–J, spanning humid marine to dry dust conditions, to represent the variability in humidity and composition in the Tel Aviv coastal environment. This classification helps constrain uncertainties related to hygroscopic growth and semivolatile losses, improving consistency between AOD and PM_2.5_ observations.

While recent global analyses (e.g., Zhu et al.^25^) have confirmed the spatial heterogeneity of the AOD-PM_2.5_ relationship across varying aerosol compositions and vertical profiles, our work advances this understanding by isolating the physical mechanisms that govern this variance. A broad body of literature has demonstrated that satellite-based PM estimation can achieve high predictive skill when AOD is combined with environmental data predictors using ML approaches. As summarized in Table 2 of Zaman et al.,^11^ studies across China, North America, Europe, and India integrate AOD products with meteorological variables, land-use parameters, and gaseous pollutants. These studies consistently show that nonlinear ML models and deep learning architectures often achieve *R* ^2^ values exceeding 0.7. Notably, most of these studies span large regions, where substantial spatial gradients in emissions, meteorology, and aerosol composition enhance the statistical contrast available to ML models. In contrast, our analysis is confined to a single metropolitan area, where spatial variability in the AOD is inherently more limited, making *R* ^2^ estimation from space more challenging. Despite this constraint, our results demonstrate that under certain aerosol layering conditions (layering types F, J, A, and E) ML can achieve *R* ^2^ 0.7–0.77. Importantly, many studies that estimate PM_2.5_ incorporate Land Use Regression (LUR) or other statistical approaches using geographic predictors (e.g., population density and road proximity). These models frequently achieve relatively high predictive accuracy, as they aim to describe human and meteorologically induced conditions at each ground monitoring station (e.g., *R* ^2^ = 0.74; *R* ^2^ = 0.85 for the EM region^36,37^ ). These models also rely on the temporal stability of spatial emission patterns and are less sensitive to dynamic atmospheric conditions. The “atmospheric” parameter is sometimes treated as the “random” effect.^9,83,84^ Our findings on vertical layering conditions offer a mechanism to enhance these models. By identifying regimes where columnar AOD is physically decoupled from the surface (e.g., “No Dust”/Type G), we provide a physical basis for weighting satellite inputs. In this regard, we suggest integrating vertical layering types as interaction terms or a categorical variable in hybrid spatiotemporal models if this type of data, or similar, is available.

Based on our results, we propose the following strategic recommendations for future studies:

- An automated predictive system should be created where the first step is to classify the layering regime (A–J) using meteorological and satellite data and the next one is to apply the appropriate, highly accurate regime-specific model. For example, for the “Lofted Dust” regime, advanced temporal features need to be incorporated, such as the use of AOD_yesterday_ as the primary predictor to account for gravitational settling time.
- Space-borne lidars such as CALIPSO, EarthCARE missions,^85^ and the future LUCE^186^ can provide aerosol layering information globally and at a greater spatial extent than that used in the current study. Generally, the horizontal representativeness of point lidar measurements varies between 50 km for turbulent boundary layers and over 200 km for stable free-tropospheric transport (e.g., dust storm).^86–89^ In addition, MAIA (Multi-Angle Imager for Aerosols)^90^ is an upcoming mission that will measure near-surface PM properties. Its accuracy across different aerosol layering conditions must be evaluated to ensure reliable characterization. Although their spatial and temporal coverage might be insufficient, they still pave the road for the first studies that estimate this effect.
- Another future work should investigate using EnMAP’s (Environmental Monitoring and Analysis Program) hyperspectral satellite to identify the aerosol physicochemical fingerprint on a pixel-by-pixel basis. This approach, we believe, will transform the point-based regime classification into a spatially continuous map. Similar work was done to identify mineralogical composition of advected dust storm.^91^

## Supplementary Material



## Notes

Relative humidity profiles as measured by the radiosonde at noon and midnight and by the lidar (Figure S1); PM_2.5_ and AOD temporal trends (Figure S2) (PDF)

## §

Mitrani Department of Desert Ecology, The Jacob Blaustein Institutes for Desert Research, Ben-Gurion University, Midreshet Ben-Gurion, 8499000, Israel
