# Supplementary material for: Vertical Aerosol Structure Matters: Improving the AOD–PM2.5 Link for Air Quality and Exposure
Source: Environ Sci Technol. 2026 May 8;60(20):14685–97. doi: 10.1021/acs.est.6c00095 (PMC13217542; doi:10.1021/acs.est.6c00095)
Supplement: Supplementary file 1 [file es6c00095_si_001.pdf]

Supporting Information for:

Vertical Aerosol Structure Matters: Improving the AOD–$PM_{2.5}$ Link for Air Quality and Exposure

Irina Rogozovsky[1,a], **Albert Ansmann**[2], **Alexandra Chudnovsky**[1]

[1]Department of Geophysics, Raymond and Beverly Sackler Faculty of Exact Sciences, Air-O lab, Tel Aviv University, Tel Aviv, 6997801, Israel

[2]Leibniz Institute for Tropospheric Research, Leipzig, 04318, Germany

[a]*Present address:* Mitrani Department of Desert Ecology, The Jacob Blaustein Institutes for Desert Research, Ben-Gurion University, Midreshet Ben-Gurion, 8499000, Israel

Corresponding Authors email address: irinar@mail.tau.ac.il, achudnov@tauex.tau.ac.il

Summary: 2 pages including 2 figures.

Table of Contents

- Figure S1: S1
- Figure S2: S2

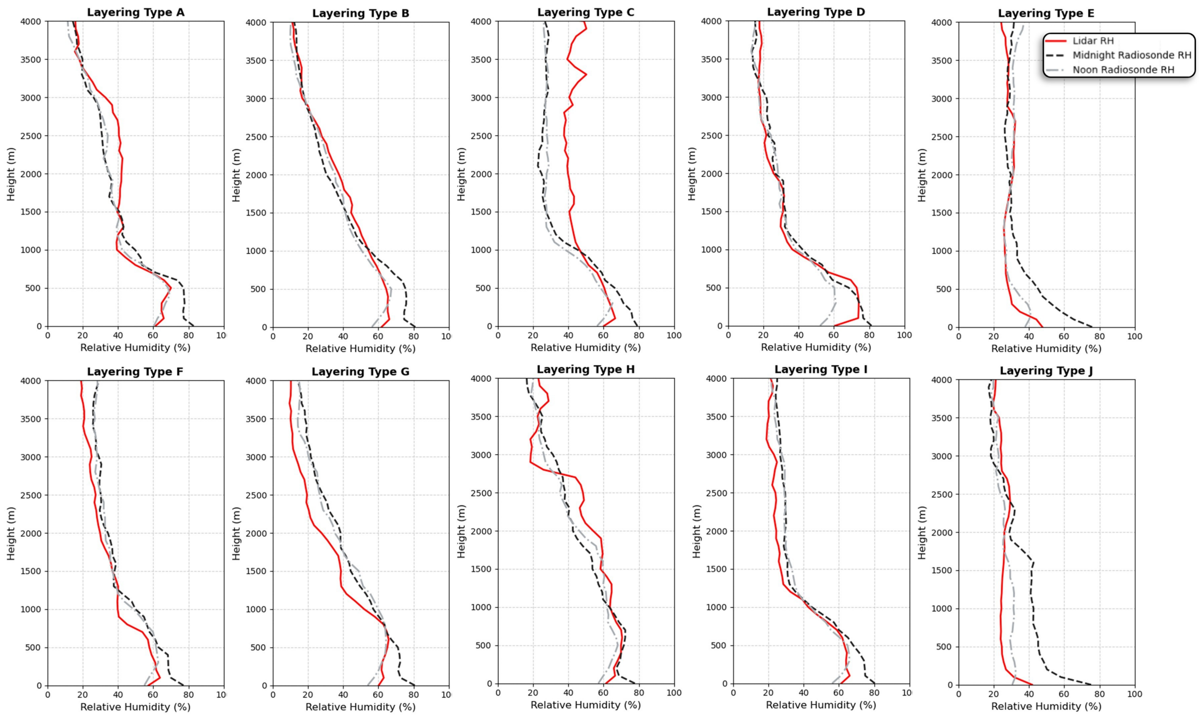

Fig. S1: Relative humidity measured by lidar (red), radiosonde at midnight (black) and noon (gray). Each panel represents one of the aerosol layering conditions (A–J).

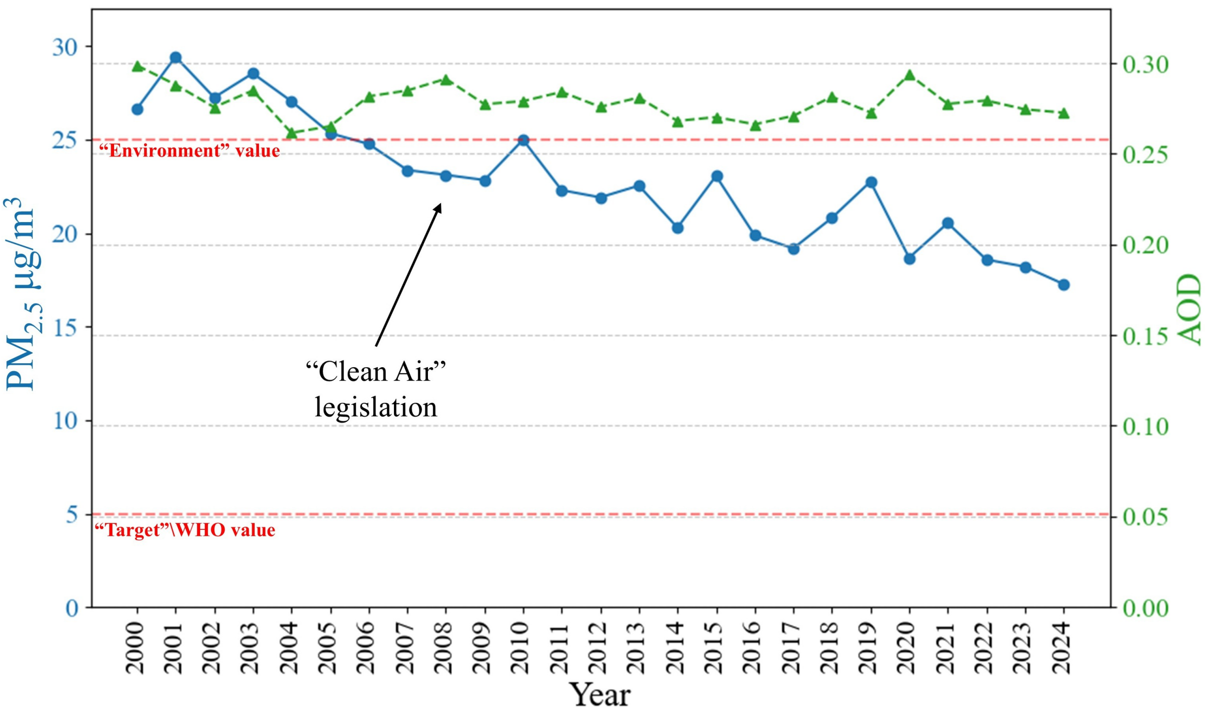

Fig. S2: $PM_{2.5}$ and MAIAC AOD time series. $PM_{2.5}$ (blue, left axis) represents annual mean concentrations calculated from 24-hour averaged measurements for each year. MAIAC AOD (green, right axis) represents annual mean AOD derived from daily satellite overpass observations. $PM_{2.5}$ shows a decreasing trend over time, whereas AOD remains relatively stable.
